# Road traffic noise frequency and prevalent hypertension in Taichung, Taiwan: A cross-sectional study

**DOI:** 10.1186/1476-069X-13-37

**Published:** 2014-05-16

**Authors:** Ta-Yuan Chang, Rob Beelen, Su-Fei Li, Tzu-I Chen, Yen-Ju Lin, Bo-Ying Bao, Chiu-Shong Liu

**Affiliations:** 1 Department of Occupational Safety and Health, College of Public Health, China Medical University, 91 Hsueh-Shih Road, Taichung 40402, Taiwan, R.O.C; 2 Institute for Risk Assessment Sciences, Utrecht University, P.O. Box 80178, 3508 TD Utrecht, The Netherlands; 3 Department of Pharmacy, College of Pharmacy, China Medical University, 91 Hsueh-Shih Road, Taichung 40402, Taiwan, R.O.C; 4 Department of Family Medicine, China Medical University Hospital, 2 Yuh-Der Road, Taichung 40447, Taiwan, R.O.C

**Keywords:** Cross-sectional study, Hypertension, Prevalence, Transportation noise

## Abstract

**Background:**

Epidemiological studies have reported the association between hypertension and exposure to road traffic noise, but the association between noise frequency characteristics is not clear. This study investigated the association between exposure to different frequency components of road traffic noise and the prevalence of hypertension in central Taiwan.

**Methods:**

We recruited 820 residents living near main roads for more than 3 years. Frequency components of traffic noise and traffic flow rates during 0900–1700 on weekdays were measured simultaneously in 2008. Multiple logistic regressions were conducted to estimate odds ratios (ORs) for diagnosed hypertension, adjusting for potential confounders and the total traffic flow rate.

**Results:**

The high-exposure group (≥ the median of noise levels [decibels, dB]) at 63 Hz, 125 Hz and 1000 Hz had ORs for hypertension of 2.77 (95% confidence interval [CI]: 1.17-6.52), 4.08 (95% CI: 1.57-10.63) and 1.98 (1.00-3.92) (95% CI: 1.00-3.92), respectively, compared to the low-exposure group (< the median of noise levels [dB]). There was an increasing trend in the prevalence of hypertension by exposure to road traffic noise at 63, 125 and 1000 Hz in all subjects and in men. Total subjects exposed to ≥ 51 dB at 125 Hz had an OR of 4.65 (95% CI = 1.46-14.83) compared to those exposed to < 47 dB.

**Conclusions:**

With the possible bias of exposure misclassification and a bias from using diagnosed hypertension, these results suggest that exposure to road traffic noise at low and hearing-sensitive frequencies may be associated with hypertension and exposure to noise at 125 Hz may have the greatest risk for hypertension.

## Background

Some studies have reported that exposure to road traffic noise is associated with myocardial infarction [1-4]. Noise exposure may induce the development of hypertension, which is an important risk factor for cardiovascular disease, and many epidemiological studies have shown the association between hypertension and road traffic noise exposure [5-12]. Road traffic noise exposure may produce hypertension via the neuroendocrine system. This exposure may cause annoyance and an emotional response in cortical and subcortical structures by interfering with communication, tasks that require high levels of concentration, relaxation, and sleep [13-15].

However, the association between hypertension and frequency components of road traffic noise is not clear. Exposure to different noise frequencies may have different effects on hypertension. Occupational noise-induced hearing loss at 4000 or 6000 Hz may be associated with hypertension in male workers [16]. One cross-sectional study has found a higher but non-significant prevalence of hypertension among male workers exposed to noise levels at frequencies of 2000, 4000 and 8000 Hz [17]. Exposure to low-frequency noise from 10 to 200 Hz has been recognized as an environmental pollutant that is associated with annoyance in previous studies [18,19]. One meta-analysis demonstrated a positive and significant association between road traffic noise annoyance and arterial hypertension [20]. To our knowledge, no study has been conducted to investigate the association between hypertension and exposure to different frequencies of road traffic noise. The association between total noise exposure and the prevalence of hypertension among inhabitants has been evaluated in a previous paper [11] and this study goes on to investigate associations with specific noise frequencies. The purpose of this study was to determine whether different frequency components of road traffic noise exposure have different associations on the prevalence of hypertension among residents in central Taiwan.

## Methods

### Study population

This cross-sectional study was conducted in Taichung, a city with a population of 1.07 million people located in central Taiwan. The recruitment and selection of study subjects have been described in detail previously [11]. Briefly, four main roads were chosen as study areas, including three roads radiated from the Taichung Station across the city and one road linked to the other roads near this station. A total of 77.3% of Taichung inhabitants live in the area that the four roads cover. To simultaneously determine road traffic noise levels and traffic flow rates, 42 sampling sites were established at 1-km intervals radiating from the station. We recruited 20 households for each sampling site along the four main roads from June to September 2008. Only one person per household was invited to participate in this study. Study subjects must have lived within a 100-m radius of these sampling sites for more than 3 years at the current address to be included in the study. The interview was carried out by four well-trained investigators in the subjects’ homes during the periods of road traffic noise measurements. Because the study area includes the shopping district and residential district in Taichung City, most participants are retailers, owners of grocery stores (who are running a business on the first floor and living on the higher ones at the current address) or housewives except some may be unemployed or retired. We excluded 20 subjects who had been living in their homes for less than 3 years. Ultimately, 820 residents were included as study subjects. The participants included 321 men (36.5 ± 13.9 years) and 499 women (35.6 ± 12.1 years). This study was reviewed and approved by the Institutional Review Board of the School of Public Health of China Medical University before the study commenced, and written informed consent was obtained from each participant.

### Questionnaire and definition of hypertension

We used a standardized questionnaire to collect personal data related to additional risk factors for hypertension. These factors included age, gender, height, weight, smoking history, alcohol consumption, tea consumption, coffee consumption, daily salt intake, exercise habits and family history of hypertension. To avoid information bias, the subjects’ lifestyle habits were defined in detail [11,16]. For instance, current smokers were defined as participants who admitted to smoking more than three times per week for at least six months. Subjects were considered to have high salt intake if they reported that they consumed more dietary salt than other study participants based on the median consumption. A family history of hypertension was positive if the subject had parents or grandparents with doctor-diagnosed hypertension. In addition, body mass index (BMI) was calculated as body weight (kg) divided by the square of the height (m^2^).

A research participant was considered to be a case of hypertension if he answered affirmatively to the question: “Have you been diagnosed with hypertension by a physician in the past while living at the current address?” Of the 820 subjects in this study, 46 cases were identified using this criterion. Accordingly, the participants were divided into a case group of 46 subjects and a control group that consisted of the remaining 774 subjects.

### Noise frequency analyses and traffic flow rates

Road traffic noise levels were measured with an octave-band analyzer (TES-1358, TES Electronic Corp., Taipei, Taiwan), which can report 1-second to 24-hour continuous equivalent sound levels (Leq) in the range of 30–130 A-weighted decibels (dBA) and time-weighted average (TWA) noise levels at frequencies of 31.5, 63, 125, 250, 500, 1000, 2000, 4000 and 8000 Hz. This equipment was calibrated with a sound-level calibrator (TES-1356, TES Electronic Corp., Taipei, Taiwan) before the noise measurements were obtained. Forty-two sampling sites were set up at 1-km intervals along each of four main roads. The sampling sites were located 1 m away from buildings and at a height of 1.5 m. Industrial hygienists measured 15-minute TWA Leq from each of the 42 sampling sites on weekdays from 0900–1700. It was impossible to measure each subject’s individual noise exposure every day; therefore, all subjects were divided into exposure groups based on the closest sampling site. The distance between the sampling site and a subject’s address ranged from 5.2 m to 67.7 m within each group. Each subject was assigned to a road traffic noise level and the frequency components that corresponded with the 8-hour TWA Leq measured at the closest site.

During the monitoring period, two research assistants assessed traffic flow rates of heavy-duty diesel trucks (HDDTs, ≥ 3.5 ton), light-duty diesel trucks (LDDTs, < 3.5 ton), light-duty gasoline vehicles (LDGVs, < 3.5 ton) and motorcycles at each of the sites. Each assistant counted two types of traffic vehicles passing in front of the sampling sites. The total traffic flow rate was the sum of motorcycles, LDGVs, LDDTs and HDDTs in this study.

### Statistical analysis

To investigate the association between exposure to frequency components of road traffic noise and the prevalence of hypertension, the Shapiro-Wilk test was used to determine the normality of the continuous variables, including age, BMI, 8-hour A-weighted equivalent sound level (LAeq 8 h) and frequency components of noise exposure. The statistical *p* values for these variables were less than 0.001 among all participants, indicating a non-normal distribution. Therefore, univariate comparisons between the case and control groups were performed using the Wilcoxon signed rank sum test for continuous variables and the Chi-square test for dichotomous variables. The Shapiro-Wilk test was also used to assess the normality of the frequency components of traffic noise and traffic flow rates at the sampling sites. The traffic flow-rate variables were not normal; therefore, non-parametric Spearman correlation coefficients were calculated to determine which frequency component was dominantly correlated with the traffic.

Continuous (i.e., 1-dB increase at each frequency) and categorical variables of road traffic noise in participants were used to examine the association with hypertension. Because participants had large variations in the noise intensity (from 19 dB at 31.5 Hz to 66 dB at 1000 Hz) and exposure ranges (from 10 dB at 125 Hz to 22 dB at 63 Hz) at nine frequencies (Additional file 1: Figure S1), the median values at different frequencies were used to separate subjects into high- and low-exposure groups with a similar number in each subgroup. For the same reason, the 820 subjects were divided into quartiles of noise exposure, stratifying by frequency, to investigate an exposure-response trend.

Logistic regressions were used to calculate odds ratios (ORs) and 95% confidence intervals (CIs) in this study. For each of the nine frequencies of traffic noise levels ranged from 31.5 Hz to 8000 Hz, the OR of self-reported hypertension in participants above vs. below the median exposure were calculated. Four variables of gender (male vs. female), age (years), BMI (kg/m^2^) and family history of hypertension (yes vs. no) were significant (all *p* values < 0.050) between the case and control groups; therefore, they were used to establish the model 2 in the analyses. Additionally, multiple logistic regressions adjusted for these variables and four important risk factors of hypertension reported in the literature, including cigarette smoking, alcohol consumption, high salt intake and physical inactivity [21,22], were established as the model 3 (main model) in this study. The extended models (i.e., model 4 and model 5) were also set up to show the results of the model 3 adjusted for the total noise exposure and the total traffic flow rate, respectively. The effect modification by sex was investigated using stratified analyses to test the interaction at nine frequencies of road traffic noise. The SAS standard package for Windows version 9.2 (SAS Institute Incorporation, Cary, North Carolina, USA) was used for statistical analyses. The significance level was set at 0.050 for all statistical tests in the present study.

## Results

Table 1 summarizes the characteristics of the 820 study participants. For simplicity, only data related to hypertensive cases are presented here. There were significant differences in age, BMI, gender and family history of hypertension between the case and control groups. Subjects in the case group were significantly older and had higher BMIs than did subjects in the control group. In addition, the case group had higher proportions of male subjects and participants with a positive family history of hypertension.

**Table 1:** Characteristics of the study participants

**Characteristics**	**Case group**^**a**^	**Control group**	**Total subjects**	***P*-value**
	**(n = 46)**	**(n = 774)**	**(n = 820)**	
Age (years)				
Mean ± SD	49.3 ± 11.6	35.2 ± 12.5	36.0 ± 12.8	<0.001^b^
Body mass index (kg/m^2^)				
Mean ± SD	25.5 ± 4.3	22.3 ± 3.5	22.5 ± 3.6	<0.001^b^
Gender				
Male (%)	25 (54.4)	296 (38.2)	321 (39.2)	0.030^c^
Current smoker				
Yes (%)	12 (26.1)	175 (22.6)	187 (22.8)	0.585^c^
Alcohol drinking				
Yes (%)	9 (19.6)	83 (10.7)	92 (11.2)	0.065^c^
Tea consumption				
Yes (%)	30 (65.2)	508 (65.6)	538 (65.6)	0.954^c^
Coffee consumption				
Yes (%)	16 (34.8)	294 (38.0)	310 (37.8)	0.664^c^
Salt intake				
High (%)	16 (34.8)	194 (25.1)	210 (25.6)	0.142^c^
Regular exercise				
Yes (%)	25 (54.4)	383 (49.5)	408 (49.8)	0.522^c^
Family history of hypertension				
Yes (%)	36 (78.3)	240 (31.0)	276 (33.7)	<0.001^c^

SD = standard deviation. ^a^Subjects had the previous diagnosis of hypertension while living at the current address. ^b^Wilcoxon signed rank sum test of the difference between the two groups. ^c^Chi-square test of the difference between the two groups.

Table 2 presents the Spearman correlations between traffic flow rates and the frequency components of road noise. All types of vehicles were significantly (all *p* values < 0.050) but moderately correlated (rho ranged from 0.31 to 0.63) with low frequency components of traffic noise and with the medium frequency at 250 Hz. Only the traffic flow rate of motorcycles was also significantly (all *p* values < 0.050) correlated with high frequency components of traffic noise and with the 500 Hz band of medium frequency components, having moderate values of correlation coefficients (rho = 0.31 to 0.49). Accordingly, the total traffic flow rate was significantly (all *p* values < 0.050) but moderately correlated (rho ranged from 0.33 to 0.64) with most frequency components except the frequencies of 1000 and 2000 Hz.

**Table 2:** Spearman correlations between frequency components of road noise levels and traffic flow rates

**Vehicle type**	**Traffic flow number**	**Median (Q1-Q3) (vehicle/hr)**	**Correlation coefficients**								
			**Low frequency**			**Medium frequency**			**High frequency**		
			**31.5 Hz**	**63 Hz**	**125 Hz**	**250 Hz**	**500 Hz**	**1000 Hz**	**2000 Hz**	**4000 Hz**	**8000 Hz**
MC	42	494 (280–704)	0.505^a^	0.594^a^	0.574^a^	0.488^b^	0.351^c^	0.279	0.312^c^	0.493^a^	0.395^a^
LDGV	42	600 (360–1032)	0.630^a^	0.592^a^	0.515^a^	0.356^c^	0.276	0.248	0.204	0.301	0.218
LDDT	42	138 (80–228)	0.398^b^	0.371^c^	0.491^b^	0.313^c^	0.110	0.162	0.147	0.132	0.093
HDDT	42	42 (24–80)	0.482^b^	0.539^a^	0.456^b^	0.350^c^	0.301	0.252	0.233	0.271	0.213
Total	42	1552 (764–2132)	0.601^a^	0.643^a^	0.586^a^	0.457^b^	0.331^c^	0.272	0.274	0.393^c^	0.340^c^

HDDT = heavy-duty diesel trucks; LDDT = light-duty diesel trucks; LDGV = light-duty gasoline vehicles; MC = motorcycles; Q1 = first quartile; Q3 = third quartile. ^a^*P* < 0.001. ^b^*P* < 0.010. ^c^*P* < 0.050.

The correlations between total noise exposure and noise levels at low, medium and high frequencies are shown in the supplementary table (Additional file 1: Table S1). Total noise levels were significantly correlated with all frequencies (all *p* values < 0.050) and the higher correlations (correlation coefficients > 0.85) were observed at low frequencies of 63, 125 and 250 Hz. Additionally, noise exposure at high frequencies of 2000, 4000 and 8000 Hz had moderate correlations with those at median frequencies of 250, 500 and 1000 Hz (rho ranged from 0.49 to 0.96, all *p* values < 0.050) but lower correlations with those at low frequencies of 63 and 125 Hz (rho ranged from 0.39 to 0.64, all *p* values < 0.050).

Table 3 shows the measurements of environmental noise exposure and noise frequency components for the two groups. The cases were exposed to the significantly higher noise levels than the controls in the 8-hour TWA Leq and at frequencies of 63, 125, 250 and 500 Hz (all *p* values < 0.050). The noise distributions of different frequency components among all participants show large variations with the highest median of 61 dB at 1000 Hz and a lowest one of 27 dB at 31.5 Hz (Additional file 1: Figure S1).

**Table 3:** Cases of hypertension and associated exposure to different noise frequencies

**Characteristics**	**Case group (n = 46)**	**Control group (n = 774)**	**Total subjects (n = 820)**	***P*-value**
LAeq 8 h (dBA)				
Median (Q1, Q3)	81 (79, 82)	79 (77, 82)	79 (77, 82)	0.016^a^
Frequency components				
31.5 Hz (dB)				
Median (Q1, Q3)	29 (26, 31)	27 (25, 30)	27 (25, 30)	0.222^a^
63 Hz (dB)				
Median (Q1, Q3)	43 (41, 45)	41 (38, 44)	41 (38, 44)	0.026^a^
125 Hz (dB)				
Median (Q1, Q3)	50 (48, 52)	49 (47, 51)	49 (47, 51)	0.032^a^
250 Hz (dB)				
Median (Q1, Q3)	55 (53, 56)	54 (52, 55)	54 (53, 55)	0.017^a^
500 Hz (dB)				
Median (Q1, Q3)	58 (57, 60)	58 (56, 59)	58 (56, 59)	0.032^a^
1000 Hz (dB)				
Median (Q1, Q3)	61 (60, 63)	61 (59, 63)	61 (59, 63)	0.070^a^
2000 Hz (dB)				
Median (Q1, Q3)	59 (57, 61)	59 (57, 61)	59 (57, 61)	0.120^a^
4000 Hz (dB)				
Median (Q1, Q3)	57 (54, 58)	56 (53, 59)	56 (53, 59)	0.458^a^
8000 Hz (dB)				
Median (Q1, Q3)	50 (48, 52)	49 (45, 52)	49 (45, 52)	0.096^a^

dB = decibel; dBA = A-weighted decibel; LAeq 8 h = A-weighted equivalent sound level at 09:00–17:00; Q1 = first quartile; Q3 = third quartile. ^a^Wilcoxon signed rank sum test of the difference between the two groups.

Table 4 summarizes the associations between the prevalence of hypertension and exposure to different frequency components of road traffic noise. Multiple logistic regression models showed that the OR for hypertension was significantly higher in the high-exposure group at 63, 125 and 1000 Hz compared to the low-exposure group after adjustment for potential confounders in both model 2 and model 3 (with all confounding factors included). Subjects exposed to ≥ 41 dB at 63 Hz, ≥ 49 dB at 125 Hz and ≥ 61 dB at 1000 Hz had ORs of 2.14, 2.51 and 1.99, respectively. Such associations were not significant after adjusting for the total noise exposure in model 4 because it had high correlations with frequency components that might cause the over-adjustment. However, the results were pronounced at frequencies of 63 and 125 Hz after controlling for the total traffic flow rate, indicating the possible interaction with other traffic pollutants. No effect modification by sex could be shown for associations between exposure to traffic noise at 63, 125 and 1000 Hz (as shown in Additional file 1: Table S3), possibly caused by the lack of power in the stratified analyses.

**Table 4:** Associations between hypertension and categorical exposure of traffic road noise stratified by different frequency components

**Frequency component**	**Categorical exposure**	**Median**	**Model 1**^**a**^	**Model 2**^**b**^	**Model 3**^**c**^	**Model 4**^**d**^	**Model 5**^**e**^
			**OR (95% CI)**	**OR (95% CI)**	**OR (95% CI)**	**OR (95% CI)**	**OR (95% CI)**
31.5 Hz	HEG vs. LEG	27 dB	1.33 (0.73-2.43)	1.17 (0.61-2.27)	1.08 (0.55-2.12)	0.52 (0.22-1.21)	1.05 (0.52-2.12)
63 Hz	HEG vs. LEG	41 dB	1.82 (0.97-3.42)	2.20 (1.10-4.39)^f^	2.14 (1.06-4.31)^f^	1.19 (0.41-3.43)	2.77 (1.17-6.52)^f^
125 Hz	HEG vs. LEG	49 dB	2.09 (1.08-4.02)^f^	2.53 (1.24-5.18)^f^	2.51 (1.21-5.21)^f^	1.73 (0.67-4.49)	4.08 (1.57-10.63)^f^
250 Hz	HEG vs. LEG	54 dB	1.48 (0.80-2.74)	1.56 (0.79-3.05)	1.50 (0.75-2.98)	0.70 (0.27-1.78)	1.52 (0.73-3.16)
500 Hz	HEG vs. LEG	58 dB	1.32 (0.73-2.39)	1.42 (0.73-2.75)	1.36 (0.69-2.67)	0.72 (0.31-1.67)	1.34 (0.68-2.66)
1000 Hz	HEG vs. LEG	61 dB	1.73 (0.95-3.15)	2.08 (1.06-4.06)^f^	1.99 (1.01-3.93)^f^	1.40 (0.64-3.05)	1.98 (1.00-3.92)^f^
2000 Hz	HEG vs. LEG	59 dB	1.52 (0.84-2.76)	1.76 (0.91-3.41)	1.73 (0.89-3.39)	1.17 (0.54-2.56)	1.73 (0.87-3.43)
4000 Hz	HEG vs. LEG	56 dB	1.67 (0.88-3.18)	1.75 (0.87-3.51)	1.74 (0.85-3.56)	1.22 (0.55-2.71)	1.73 (0.84-3.57)
8000 Hz	HEG vs. LEG	49 dB	1.63 (0.88-3.01)	1.68 (0.86-3.29)	1.71 (0.86-3.39)	1.52 (0.75-3.04)	1.72 (0.87-3.42)

dB = decibel; HEG = high exposure group (≥ the median); LEG = low exposure group (< the median); OR = odds ratio; 95% CI = 95% confidence interval. ^a^Simple logistic regression model. ^b^Multiple logistic regression models adjusted for significant factors between the case and control groups (such as age, gender, body mass index and family history of hypertension). ^c^Multiple logistic regression models adjusted for all variables in model 2 and important risk factors of hypertension identified in the literature (i.e., current smoking, alcohol consumption, salt intake and physical inactivity). ^d^Model 3 adjusted for the total noise exposure. ^e^Model 3 adjusted for the total traffic flow rate. ^f^*P* < 0.050.

**Figure 1:**
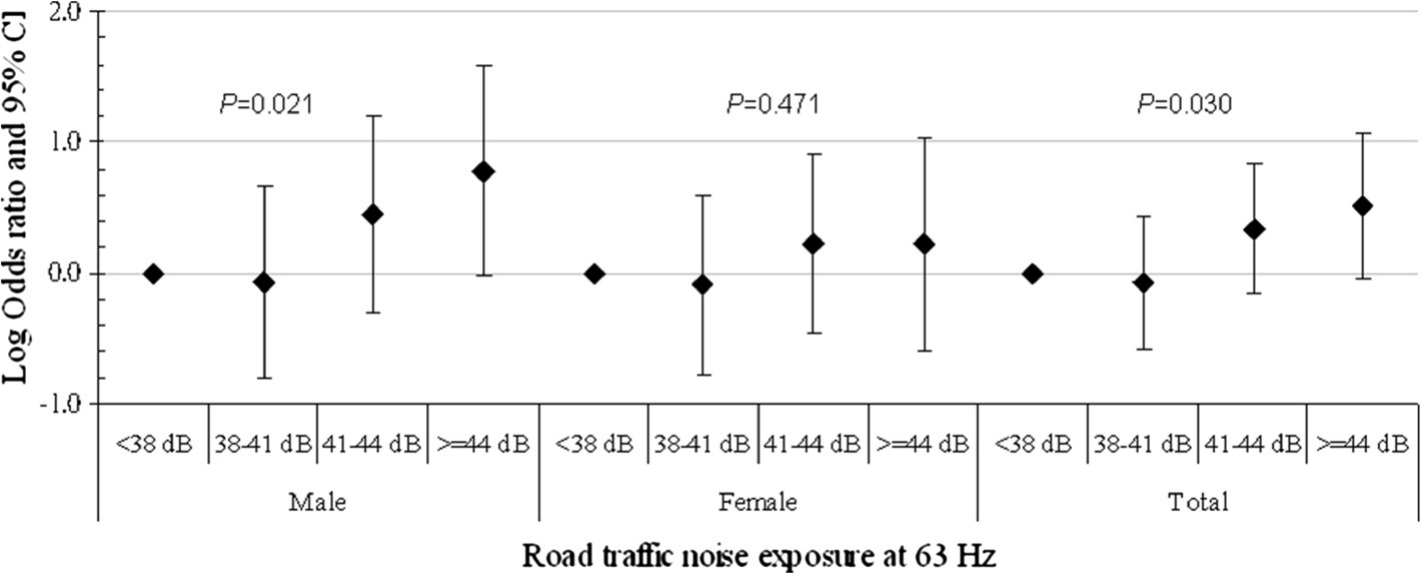
**The association between hypertension and road traffic noise frequency at 63 Hz for study subjects.** The analysis was adjusted for gender, age, body mass index, smoking status, alcohol consumption, salt intake, physical activity, family history of hypertension and the total traffic flow rate.

**Figure 2:**
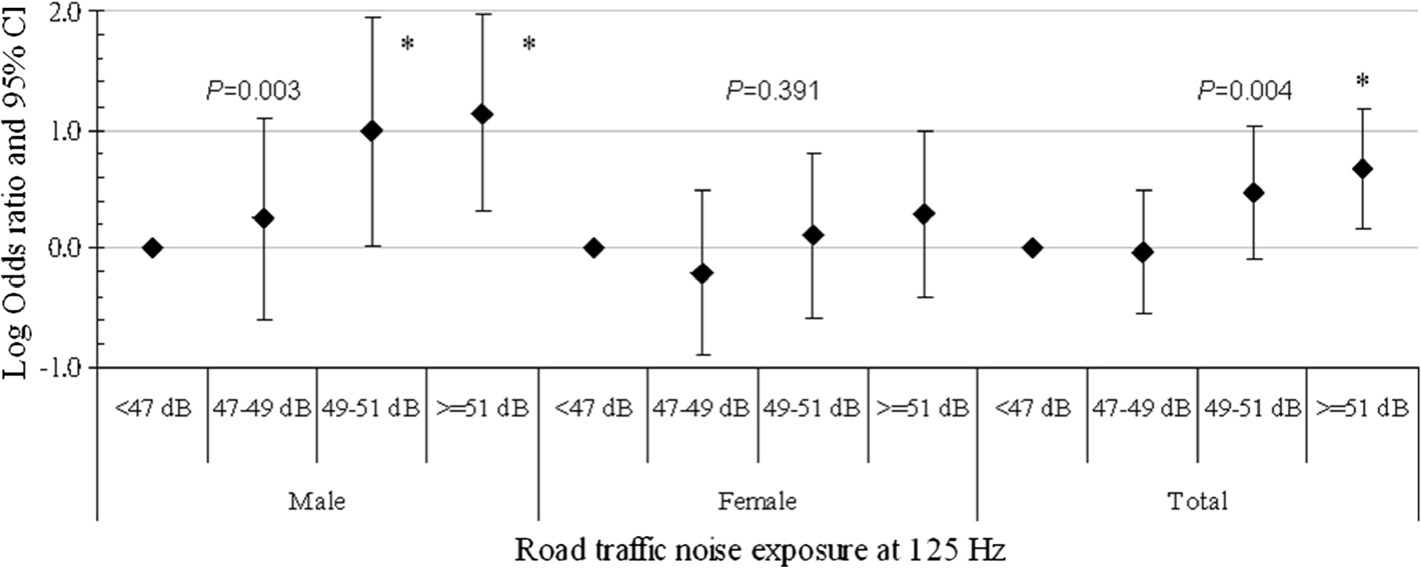
**The association between hypertension and road traffic noise frequency at 125 Hz for study subjects.** The analysis was adjusted for gender, age, body mass index, smoking status, alcohol consumption, salt intake, physical activity, family history of hypertension and the total traffic flow rate. *P < 0.05.

**Figure 3:**
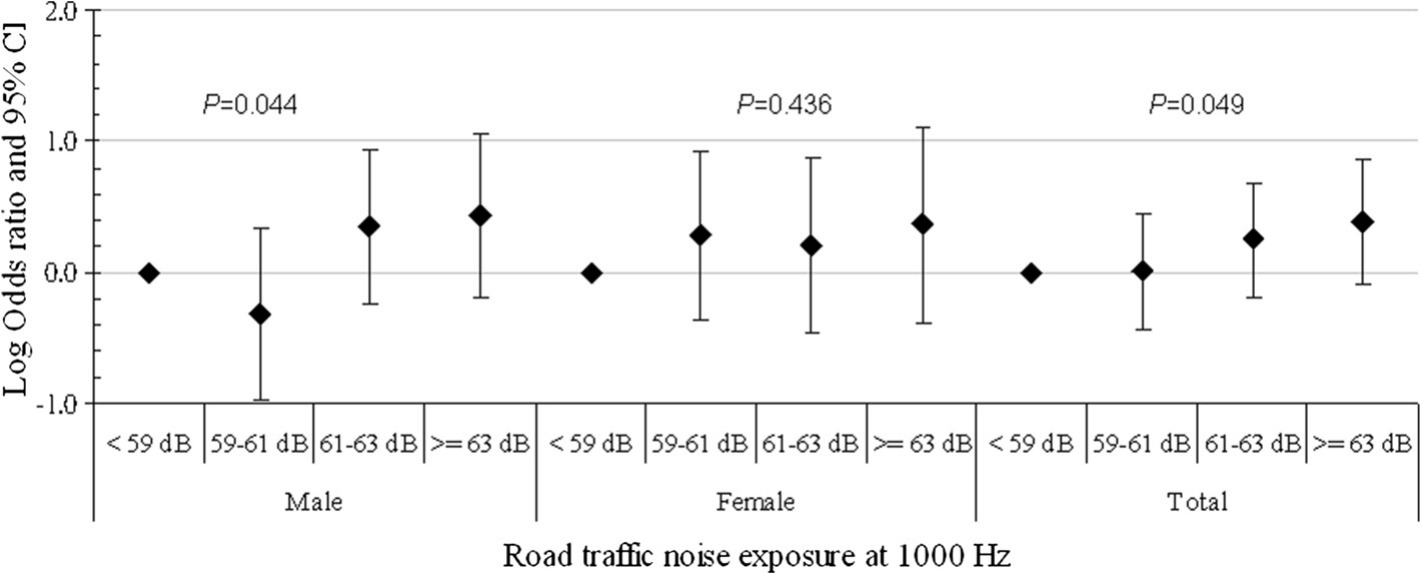
**The association between hypertension and road traffic noise frequency at 1000 Hz for study subjects.** The analysis was adjusted for gender, age, body mass index, smoking status, alcohol consumption, salt intake, physical activity, family history of hypertension and the total traffic flow rate.

To investigate the exposure-response association, the relative prevalence of hypertension is shown for different quartiles of road traffic noise at different frequency bands of the noise spectrum. Because the high-exposure group at frequencies of 63, 125 and 1000 Hz had a significantly higher OR for hypertension, road traffic noise levels at these frequencies were analyzed. Figure 1, 2, 3 show the logarithmic ORs and 95% CIs for the prevalence of hypertension in four categories of road traffic noise exposure at different frequencies. There were significantly increasing trends between exposure to road traffic noise at 63 Hz in Figure 1, 125 Hz in Figure 2 and 1000 Hz in Figure 3 and the prevalence of hypertension among total subjects after adjustment for potential confounders and the total traffic flow rate. Subjects exposed to road traffic noise ≥ 51 dB at 125 Hz had an OR of 4.65 (95% CI = 1.46-14.83) compared with those exposed to < 47 dB at 125 Hz after controlling for potential confounders.

The trends between noise exposure at 63 Hz, 125 Hz and 1000 Hz and hypertension were also observed significantly in men, but not in women (all *p* values > 0.050), although the direction of effects was similar (Figure 1, 2, 3). In addition, male subjects exposed to 125-Hz noise levels ≥ 51 dB and those exposed to 125-Hz noise levels of 49–51 dB had ORs of 13.80 (95% CI = 2.02-94.18) and 9.75 (95% CI = 1.06-89.75) compared to men exposed to 125-Hz noise levels < 47 dB after adjusting for potential confounders.

## Discussion

Our study is the first to show the association between the prevalence of hypertension and frequency components of road traffic noise. We found that residents exposed to high levels of road traffic noise at 63, 125 and 1000 Hz had a significantly higher risk of hypertension. The findings at 63 and 125 Hz support the hypothesis that exposure to low-frequency (10–200 Hz) traffic noise may produce hypertension via the neuroendocrine system by inducing annoyance at cortical or subcortical structures [13-15,18,19]. In addition, all types of traffic were significantly correlated with low-frequency noise exposure. Motorcycles, the dominant source of traffic noise in central Taiwan [11], had the highest correlation coefficients at 63 and 125 Hz. One possible explanation for the higher risk observed at 1000 Hz might be that subjects with normal hearing have prominent hearing sensitivity around 1000 Hz [23]. Another explanation is that noise exposure greater than 30 dB at 1000 Hz was reported to have 100% auditory brainstem responses in an animal study [24]. However, we did not observe the significant association between the prevalence of hypertension and noise exposure at other medium components of 250 and 500 Hz. The inconsistent results at medium frequencies might be that the human auditory system is particularly sensitive to noise exposure at 1000 Hz. Therefore, this study suggests that exposure to road traffic noise of different frequencies may have differential influence on the prevalence of hypertension.

In addition, we found a significant trend between road traffic noise exposure at 125 Hz and the prevalence of hypertension. This exposure-response pattern observed only in men was consistent with findings of previous studies [8,9,11]. In contrast, the significantly higher risk of hypertension associated with 5-dBA increases in road traffic noise was seen only in women in a community-based study [6]. One panel study showed higher 5-dBA-induced increases in systolic and diastolic blood pressures in women compared to men [25]. The difference in the prevalence of hypertension between men and women could be the result of differences in sensitivity to frequency components of road traffic noise. Future studies should be conducted to compare the effects of noise frequency on blood pressure in men and women.

The results of this study indicate that different frequency characteristics of traffic noise may have different threshold values on the risk of hypertension. We found that exposure to 125-Hz noise had the largest odds ratio on the prevalence of hypertension. Participants were assigned to the high-exposure group if they were exposed to noise levels higher than the median value of noise levels for each frequency. When adjusting for the same confounders in multiple logistic regression models (model 3), the high-exposure group had an OR of 2.15 for all frequencies in a previous study [11] compared with the findings in this study that they had an OR of 2.51 at 125 Hz, 2.14 at 63 Hz and 1.99 at 1000 Hz, respectively. In addition, per 1-dB increase in noise exposure had an OR of 1.22 (95% CI = 1.04-1.42; *p* = 0.012) at 125 Hz, 1.13 (95% CI = 0.99-1.29; *p* = 0.070) at 1000 Hz, 1.10 (95% CI = 1.02-1.18; *p* = 0.011) at 63 Hz and 1.18 (95% CI = 1.04-1.33; *p* = 0.010) for LAeq 8 h while controlling for the same confounders in the model 3.

We also found a high correlation (*r* = 0.66, *p* < 0.001) between noise exposure and total traffic flow rate that was consistent with the results of previous studies. One previous study measured traffic noise and NO_2_ in Canada (*r* = 0.53) [26] and another study measured NO_2_ and modeled traffic noise in Spain (*r* = 0.62) [27]. This correlation may be due to the study design, which measured noise levels and traffic flow rates at sampling sites along main roads but not background locations. In contrast, moderate correlations between measured traffic noise and NO_2_ (*r* = 0.32) in Madrid, Spain [28] and modeled traffic noise and measured traffic intensities (*r* = 0.30) in the Netherlands [29] have been reported. However, the high correlation in this study makes it hard to disentangle the effects of exposure to noise from those of exposure to air pollutants.

The present study was strengthened by classifying subjects based on results of environmental measurements to prevent non-differential misclassification of exposure that might bias the risk estimate towards the null value [30]. In addition, monitoring different frequency components of road traffic noise was more powerful to evaluate the association between noise frequency characteristics and the prevalence of hypertension.

However, some limitations of this study should be considered. First, the use of a questionnaire to identify subjects with hypertension may cause information bias, although many studies have adopted the same approach [6,7,9,10]. Using a self-reported history of hypertension diagnosed by a doctor has a sensitivity ranging from 33.3% to 71% and a specificity of 91%-96% compared to obtaining blood pressure measurements [31,32]. However, the sensitivity may vary by demographic group and age.

Second, blood pressure measurements were not collected during the household interview. Failing to obtain measurements may have underestimated the prevalence of hypertension and have the low prevalence of hypertension (i.e., 5.6%) in this study. This underestimation would be equally distributed between the high- and low-exposure groups; therefore, this non-differential misclassification would bias the results toward the null value. This bias might cause the higher but non-significant results in some frequency components of road traffic noise exposure.

Third, a cross-sectional design restricts the causal inferences that can be made about the effect of noise frequency components and hypertension. The information about full residential histories before living at the current address for which hypertension was diagnosed was not available that limited our ability to elaborate on the between-group differences, even though all participants were required to live at their current address for more than 3 years.

Fourth, our analyses did not include adjustment for socio-economic status. Residents living near the four main roads within 100 meter for more than 3 years were assumed to have a similar socio-economic status.

Fifth, the adjustment for occupational noise exposure was lacking in the statistical analyses. Since we conducted both the interview and the measurements of road traffic noise at the subjects’ homes during the daytime (0900–1700) on weekdays, exposure to higher levels of occupational noise might not be a main concern for housewives and unemployed participants except for some retirees. For retailers or owners of grocery stores (i.e., running a business and living in the same building), we assume that they have been exposed to both road traffic noise and commercial noise during the studying periods. Therefore, only using measurements of road traffic noise at daytime on weekdays may underestimate their noise levels, producing the possible bias of exposure misclassification in this study.

Sixth, associations could be the chance findings because we did multiple adjustments in the statistical analyses. As the results were in agreement with the possible mechanism of low-frequency noise-induced annoyance to develop hypertension [18-20], we believe that these are true associations.

Seventh, traffic-related air pollution was not considered in this study. Because we observed a high correlation (*r* = 0.66) between noise exposure and the total traffic flow rate and traffic-related air pollutants (i.e., nitrogen dioxide and nitrogen oxides) were highly correlated to daily traffic load in previous studies [27,33], the correlation was too high to do a meaningful analysis. However, when the total traffic flow rate was used as a surrogate of traffic-related air pollution in the multiple logistic regression models (i.e., model 5), the results at 63 Hz and 125 Hz were greater than those shown in the main models (i.e., model 3).

Eighth, this study had no really contrasting exposure (all come from the same source) but only the different levels to explore associations between the prevalence of hypertension and road traffic noise exposure at different frequency components. Future studies should be conducted to have the ideal samples that subjects exposure to the same LAeq but sources with different frequency spectra.

Finally, the noise measurements taken represent the short-term rather than the long-term exposure to road traffic noise. Previous studies have demonstrated that short-term noise levels may be a good indicator of long-term noise levels [26,34]; therefore, we used 15-min TWA Leq at 42 selected sampling sites to investigate the association between frequency components of road traffic noise and hypertension. In addition, indoor noise levels may differ from outdoor measurements due to the characteristics of houses, including the thickness and density of walls and the presence of open or closed windows. The boundaries between the noise source and the subjects can modify the frequency exposure. It is well known that the sound insulation performance of dwellings is generally poor at low frequencies. These limitations should be noted and avoided in future studies.

## Conclusions

This study showed an association between the prevalence of hypertension and road traffic noise exposure at low frequencies and also at mid-level frequencies to which the human auditory system is known to be sensitive. The noise-control measures for low-frequency components are recommended to improve the health of residents living near the main roads. Subjects exposed to road traffic noise at 125 Hz had the highest OR for hypertension and an exposure-response trend was found in all subjects and in men. Effects of road traffic noise exposure at different frequencies and the gender-difference on hypertension should be considered in future studies.

## Abbreviations

dB: Decibel; dBA: A-weighted decibel; LAeq: A-weighted equivalent sound level; Leq: Equivalent sound level; TWA: Time-weighted average.

## Competing interests

The authors declare that they have no competing interests.

## Authors’ contributions

TYC, SFL, BYB and CSL conceived and designed the study. TYC and RB analyzed the data and wrote the paper. SFL, TIC, YJL, BYB and CSL contributed materials and analysis tools. SFL, TIC, YJL, BYB and CSL critically revised the manuscript. All authors of this paper have read and approved the final manuscript.

## Supplementary Material

Additional file 1 Road traffic noise frequency and prevalent hypertension in Taichung, Taiwan: a cross-sectional study. Click here for file
