# Supplementary material for: Road traffic noise frequency and prevalent hypertension in Taichung, Taiwan: A cross-sectional study
Source: Environ Health. 2014 May 16;13:37. doi: 10.1186/1476-069X-13-37 (PMC4038380; doi:10.1186/1476-069X-13-37)
Supplement: Additional file 1 — Road traffic noise frequency and prevalent hypertension in Taichung, Taiwan: a cross-sectional study. [file 1476-069X-13-37-S1.doc]

**Additional file 1: Road traffic noise frequency and prevalent hypertension in Taichung, Taiwan: a cross-sectional study**

**Figure S1.** Distributions of road traffic noise (LAeq 8h) for total noise and different frequency components


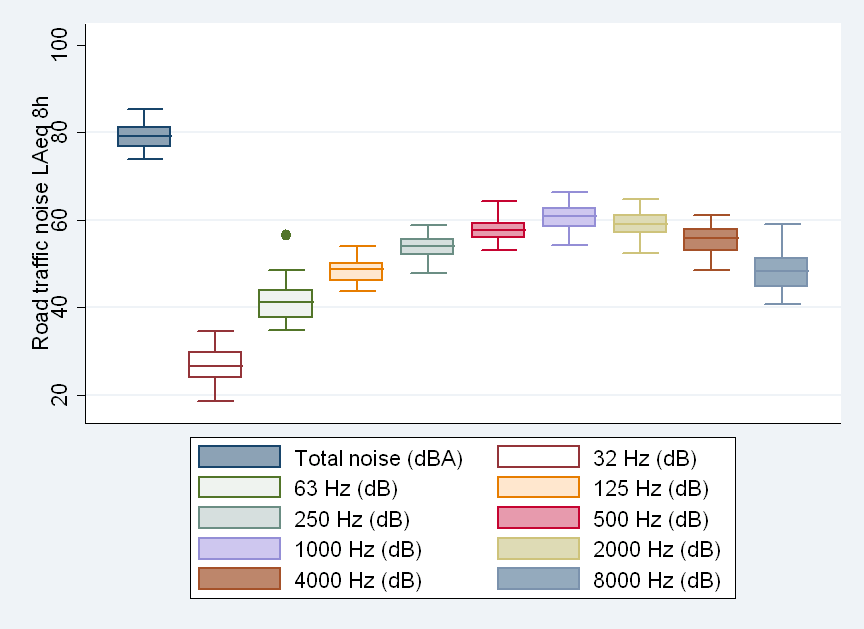


dB = decibel; dBA = A-weighted decibel; LAeq 8 h = A-weighted equivalent sound level at 09:00－17:00.

**Table S1.** Spearman’s ρ correlations of road noise levels between total noise and specific frequency components

| Frequency type | Number | Total noise | Correlation coefficients | | | | | | | | | | |
| --- | --- | --- | --- | --- | --- | --- | --- | --- | --- | --- | --- | --- | --- |
|  |  |  | Low frequency | | |  | Medium frequency | | |  | High frequency | | |
|  |  |  | 31.5 Hz | 63 Hz | 125 Hz |  | 250 Hz | 500 Hz | 1000 Hz |  | 2000 Hz | 4000 Hz | 8000 Hz |
| Total noise | 42 | 1 | 0.720^a^ | 0.929^a^ | 0.870^a^ |  | 0.865^a^ | 0.774^a^ | 0.692^a^ |  | 0.714^a^ | 0.645^a^ | 0.452^b^ |
| 31.5 Hz | 42 | - | 1 | 0.662^a^ | 0.663^a^ |  | 0.427^b^ | 0.306^c^ | 0.225 |  | 0.218 | 0.243 | 0.129 |
| 63 Hz | 42 | - | - | 1 | 0.839^a^ |  | 0.775^a^ | 0.687^a^ | 0.601^a^ |  | 0.644^a^ | 0.553^a^ | 0.416^b^ |
| 125 Hz | 42 | - | - | - | 1 |  | 0.840^a^ | 0.606^a^ | 0.552^a^ |  | 0.609^a^ | 0.520^a^ | 0.393^b^ |
| 250 Hz | 42 | - | - | - | - |  | 1 | 0.878^a^ | 0.811^a^ |  | 0.838^a^ | 0.673^a^ | 0.494^a^ |
| 500 Hz | 42 | - | - | - | - |  | - | 1 | 0.927^a^ |  | 0.924^a^ | 0.779^a^ | 0.560^a^ |
| 1000 Hz | 42 | - | - | - | - |  | - | - | 1 |  | 0.962^a^ | 0.741^a^ | 0.530^a^ |
| 2000 Hz | 42 | - | - | - | - |  | - | - | - |  | 1 | 0.795^a^ | 0.614^a^ |
| 4000 Hz | 42 | - | - | - | - |  | - | - | - |  | - | 1 | 0.902^a^ |
| 8000 Hz | 42 | - | - | - | - |  | - | - | - |  | - | - | 1 |

^a^*P* < 0.001. ^b^*P* < 0.010. ^c^*P* < 0.050.

**Table S2.** Associations between hypertension and continuous exposure of traffic road noise stratified by different frequency components

| **Frequency component** | **Continuous exposure** | **Model 1**^a^ |  | **Model 2**^b^ |  | **Model 3**^c^ |  | **Model 4^d^** |  | **Model 5**^e^ |
| --- | --- | --- | --- | --- | --- | --- | --- | --- | --- | --- |
|  |  | **OR (95% CI)** |  | **OR (95% CI)** |  | **OR (95% CI)** |  | **OR (95% CI)** |  | **OR (95% CI)** |
| 31.5 Hz | 1 dB increase | 1.06 (0.97-1.16) |  | 1.06 (0.96-1.18) |  | 1.06 (0.95-1.17) |  | 0.91 (0.79-1.06) |  | 1.06 (0.94-1.19) |
| 63 Hz | 1 dB increase | 1.07 (1.01-1.13)^f^ |  | 1.10 (1.03-1.18)^f^ |  | 1.10 (1.02-1.18)^f^ |  | 1.03 (0.88-1.21) |  | 1.15 (1.05-1.27)^f^ |
| 125 Hz | 1 dB increase | 1.14 (1.01-1.30)^f^ |  | 1.23 (1.05-1.43)^f^ |  | 1.22 (1.04-1.42)^f^ |  | 1.12 (0.89-1.40) |  | 1.25 (1.06-1.48)^f^ |
| 250 Hz | 1 dB increase | 1.20 (1.05-1.37)^f^ |  | 1.28 (1.08-1.51)^f^ |  | 1.28 (1.08-1.51)^f^ |  | 1.21 (0.94-1.56) |  | 1.29 (1.08-1.54)^f^ |
| 500 Hz | 1 dB increase | 1.12 (1.00-1.25)^g^ |  | 1.21 (1.05-1.40)^f^ |  | 1.20 (1.04-1.39)^f^ |  | 1.11 (0.92-1.35) |  | 1.20 (1.04-1.39)^f^ |
| 1000 Hz | 1 dB increase | 1.10 (0.98-1.22) |  | 1.14 (1.00-1.30)^f^ |  | 1.13 (0.99-1.29)^g^ |  | 1.04 (0.89-1.22) |  | 1.13 (0.99-1.29)^g^ |
| 2000 Hz | 1 dB increase | 1.12 (0.99-1.25)^g^ |  | 1.17 (1.02-1.35)^f^ |  | 1.16 (1.01-1.34)^f^ |  | 1.07 (0.91-1.27) |  | 1.16 (1.01-1.34)^f^ |
| 4000 Hz | 1 dB increase | 1.02 (0.93-1.12) |  | 1.03 (0.93-1.14) |  | 1.02 (0.92-1.13) |  | 0.95 (0.85-1.07) |  | 1.02 (0.91-1.13) |
| 8000 Hz | 1 dB increase | 1.04 (0.99-1.10) |  | 1.06 (0.99-1.13)^g^ |  | 1.06 (0.99-1.13)^g^ |  | 1.05 (0.98-1.13) |  | 1.06 (0.99-1.13)^g^ |

dB = decibel; OR = odds ratio; 95% CI = 95% confidence interval. ^a^Simple logistic regression model. ^b^Multiple logistic regression models adjusted for significant factors between the case and control groups (such as age, gender, body mass index and family history of hypertension). ^c^Multiple logistic regression models adjusted for all variables in model 2 and important risk factors of hypertension identified in the literature (i.e., current smoking, alcohol consumption, salt intake and physical inactivity). ^d^Model 3 adjusted for the total noise exposure. ^e^Model 3 adjusted for the total traffic volume. ^f^*P* < 0.050. ^g^*P* < 0.100.

**Table S3**. Associations between hypertension and road traffic noise stratified by sex at different frequency components.

| **Frequency component** | **Effect modifier** | **Model 3^a^** | ***P*_value for interaction** |
| --- | --- | --- | --- |
|  |  | **OR (95% CI)** |  |
| 31.5 Hz | Male | 2.79 (0.97-8.02) | 0.014 |
| 31.5 Hz | Female | 0.48 (0.17-1.32) |  |
| 63 Hz | Male | 2.59 (0.93-7.17) | 0.701 |
| 63 Hz | Female | 1.89 (0.70-5.14) |  |
| 125 Hz | Male | 3.48 (1.14-10.68)^b^ | 0.358 |
| 125 Hz | Female | 1.89 (0.69-5.24) |  |
| 250 Hz | Male | 3.32 (1.10-10.04)^b^ | 0.038 |
| 250 Hz | Female | 0.78 (0.29-2.05) |  |
| 500 Hz | Male | 2.84 (1.07-7.52)^b^ | 0.021 |
| 500 Hz | Female | 0.67 (0.24-1.87) |  |
| 1000 Hz | Male | 3.50 (1.29-9.49)^b^ | 0.094 |
| 1000 Hz | Female | 1.21 (0.45-3.21) |  |
| 2000 Hz | Male | 1.32 (0.51-3.38) | 0.642 |
| 2000 Hz | Female | 2.09 (0.79-5.55) |  |
| 4000 Hz | Male | 1.83 (0.67-4.99) | 0.774 |
| 4000 Hz | Female | 1.72 (0.61-4.84) |  |
| 8000 Hz | Male | 2.13 (0.82-5.55) | 0.572 |
| 8000 Hz | Female | 1.44 (0.54-3.84) |  |

OR = odds ratio; 95% CI = 95% confidence interval. ^a^Multiple logistic regression models adjusted for significant factors between the case and control groups (such as age, gender, body mass index and family history of hypertension) and important risk factors of hypertension identified in the literature (i.e., current smoking, alcohol consumption, salt intake and physical inactivity). ^b^*P* < 0.050.
